# Phylogenetic analyses of chikungunya virus among travelers in Rio de Janeiro, Brazil, 2014-2015

**DOI:** 10.1590/0074-02760160004

**Published:** 2016-05

**Authors:** Liliane Costa Conteville, Louise Zanella, Michel Abanto Marín, Ana Maria Bispo de Filippis, Rita Maria Ribeiro Nogueira, Ana Carolina Paulo Vicente, Marcos César Lima de Mendonça

**Affiliations:** 1 Fundação Oswaldo Cruz, Instituto Oswaldo Cruz, Laboratório de Genética Molecular de Microrganismos, Rio de Janeiro, RJ, Brasil; 2 Fundação Oswaldo Cruz, Instituto Oswaldo Cruz, Laboratório de Flavivírus, Rio de Janeiro, RJ, Brasil

**Keywords:** chikungunya virus, Asian genotype genome, Brazil

## Abstract

Chikungunya virus (CHIKV) is a mosquito-borne pathogen that emerged in Brazil by late 2014. In the country, two CHIKV foci characterized by the East/Central/South Africa and Asian genotypes, were established in North and Northeast regions. We characterized, by phylogenetic analyses of full and partial genomes, CHIKV from Rio de Janeiro state (2014-2015). These CHIKV strains belong to the Asian genotype, which is the determinant of the current Northern Brazilian focus, even though the genome sequence presents particular single nucleotide variations. This study provides the first genetic characterisation of CHIKV in Rio de Janeiro and highlights the potential impact of human mobility in the spread of an arthropod-borne virus.

Chikungunya virus (CHIKV) is a mosquito-borne pathogen that belongs to the genus *Alphavirus*, family *Togaviridae*, endemic in parts of Africa, Southeast Asia and on the Indian subcontinent*.* It usually produces a non-fatal febrile illness in humans, associated with rash and severe arthralgia (Powers & Logue 2007), and occasional neurological manifestations in children (Robin et al. 2008).

The first autochthonous CHIKV case in the Americas occurred in the Caribbean (Island of Saint Martin) in late 2013 (CDC 2014). After this event, the presence of competent vectors and the intense travel of people led to the establishment of autochthonous CHIKV cases in South American countries, besides Argentina, Chile and Uruguay (Carbajo & Vezzani 2015, PAHO/WHO 2015). In Brazil, imported cases have been reported since June 2014. By September 2014, local transmission of the Asian genotype, the one circulating in the Caribbean, was confirmed in Amapá, northern edge of Brazil. A week later, the East/Central/South African (ECSA) genotype, previously undetected in the Americas, emerged in Bahia state, North-eastern Brazil. Since then, more than 25 thousand suspected CHIKV cases were registered in Brazil (MS 2016).

Until November 2015, Rio de Janeiro state, located in the Southeast of Brazil, had only registered imported CHIKV cases (MS 2016). It is 3,000 km and 1,200 km apart from Amapá and Bahia states, respectively; the current foci of CHIKV in the country. Rio de Janeiro state was predicted to be one of the 35 municipalities with higher risk of CHIKV establishment due to importation from the North and Northeast Brazilian foci (Nunes et al. 2015). By December 2015, the first autochthonous cases were detected in the state (MS 2016).

Here, we performed a phylogenetic analysis of four CHIKV identified in the Rio de Janeiro state in 2014-2015. These CHIKV strains were from individuals with recent travel history to the Caribbean region (three of them were Brazilians with recent travel history to Curacao, Barbados and Dominican Republic, while the other patient is a Venezuelan who came to Brazil). Their main clinical manifestations were fever, arthralgia and exanthema. Whole-genome sequencing was performed on an Illumina HiSeq 2500 system (Oswaldo Cruz Foundation, high-throughput sequencing platform) using 2 x 100 bp paired-end reads generated with Nextera XT libraries. Bioinformatic analyses allowed the recovery of nearly complete genome of the CHIKV virus from the 2015 case (RJ/CHIKV/2015). E1 gene sequences were recovered by PCR and Sanger sequencing from other three 2014 cases. The sequences were submitted to GenBank under accession number KU355832-KU355835. Phylogenetic trees were constructed using Neighbor-Joining method and was evaluated by thousand bootstrap replicates.

The phylogenetic analysis of full-length genomes reveals that the RJ/CHIKV/2015 belongs to the Asian genotype (97-99% identity) and clusters together with other Brazilian imported cases - Guadalupe to Belém, Pará and Dominican Republic to Recife, Pernambuco - and an autochthone case identified in Amapá (middle 2014), as well as with strains from the Caribbean and Mexico (Figure, panel A). Considering this set of genomes, RJ/CHIKV/2015 presents eight unique single nucleotide variations. Four are nonsynonymous: P156S in the methyl-transferase domain and R1307I in the nsP1 C-terminal domain; R1806Q in the nsP3 hypervariable region; and K546R in the B-cell epitope of the E2 protein. As most CHIKV strains, RJ/CHIKV/2015 possess the opal stop codon (TGA) located at the C-terminal of the nsP3 protein, which has been associated with enhanced CHIKV replication (Chen et al. 2013).

**Figure:**
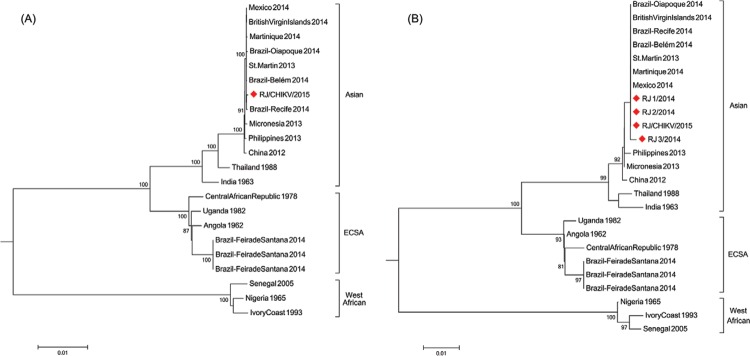
Phylogenetic trees based on full-length genome (A) and partial E1 gene (B) constructed with Neighbor-Joining approach. Sequences derived from this study are labeled by gray diamond symbols. Numbers besides internal branches indicate bootstrap values based on 1,000 replicates. Scale bar means base substitutions per site. RJ 1/2014: Venezuelan patient who came to Brazil and had the onset symptoms in 26 September 2014. RJ 2/2014: Brazilian patient with recent travel history to Barbados who had the onset symptoms in 30 September 2014. RJ 3/2015: Bra- zilian patient with recent travel history to Dominican Republic and had the onset symptoms in 22 June 2014. RJ/CHIKV/2015 is from a Brazilian patient who had the onset symptoms in 3 January 2015, after returning from the Curacao Island.

Phylogenetic analysis using partial E1 (435 bp) of the all four imported Rio de Janeiro CHIKV showed that the other three strains also belong to the same Asian genotype cluster (Figure, panel B). The E1 sequences from the Rio de Janeiro travelers are identical, except by T/C synonymous substitution in the RJ_3/2014 strain. Moreover, all of them present Alanine in the position E1-226, as the E1 gene from Asian genotype strains analysed so far, and therefore does not have the mutation that increases CHIKV transmission by *Aedes albopictus* mosquitoes (Tsetsarkin et al. 2007).

This study provides original genomic information of non-autochthonous CHIKV strains identified in travelers coming from the Caribbean region to Rio de Janeiro. This is the second most populous metropolitan area in Brazil and the primary national and international tourist attraction city of the country. Severe outbreaks caused by other arboviruses, Dengue and Zika virus, sharing the same mosquito vector as CHIKV have been oc- curring in the country as well in Rio de Janeiro (MS 2016). Our results highlight the importance of a genetic surveillance system. So far more than 25 thousand cases have been reported in Brazilian regions, and both the Asian and ECSA genotypes could be circulating in the country (Faria et al. 2016, MS 2016).

## References

[B1] Carbajo AE, Vezzani D (2015). Waiting for chikungunya fever in Argentina: spatio-temporal risk
maps. Mem Inst Oswaldo Cruz.

[B2] CDC (2014). Chikungunya virus.

[B3] Chen KC, Kam Y-W, Lin RTP, Ng MM-L, Ng LF, Chu JJH (2013). Comparative analysis of the genome sequences and replication profiles
of chikungunya virus isolates within the East, Central and South African (ECSA)
lineage. Virol J.

[B4] Faria NR, Lourenço J, Cerqueira EM, Lima MM, Pybus O, Alcantara LC (2016). Epidemiology of chikungunya virus in Bahia, Brazil,
2014-2015. PLoS Currents Outbreaks.

[B5] MS (2016). Boletim Epidemiológico.

[B6] Nunes MRT, Faria NR, Vasconcelos JM, Golding N, Kraemer MU, Oliveira LF (2015). Emergence and potential for spread of chikungunya virus in
Brazil. BMC Med.

[B7] PAHO/WHO (2015). Number of reported cases of chikungunya fever in the Americas.

[B8] Powers AM, Logue CH (2007). Changing patterns of chikungunya virus: re-emergence of a zoonotic
arbovirus. J Gen Virol.

[B9] Robin S, Ramful D, Le Seach F, Jaffar-Bandjee MC, Rigou G, Alessandri JL (2008). Neurologic manifestations of pediatric chikungunya
infection. J Child Neurol.

[B10] Tsetsarkin KA, Vanlandingham DL, McGee CE, Higgs S (2007). A single mutation in chikungunya virus affects vector specificity and
epidemic potential. PLoS Pathog.

